# Atorvastatin Reduces Alcohol‐Related Cardiac and Liver Tissue Damage, Oxidative Stress, Inflammation, Apoptosis, and Thiol‐Disulfide Imbalance in Rats

**DOI:** 10.1002/jbt.71036

**Published:** 2026-07-21

**Authors:** Cumaali Demirtas, Şahhan Kılıç, Mert Babaoğlu, Süha Asal, Elif Gökçe Tenekeci, Hakan Beyaztaş, Eray Metin Guler, Salime Pelin Erguven, Kubra Sevgin

**Affiliations:** ^1^ Hamidiye Health Sciences Institute University of Health Sciences Istanbul Turkiye; ^2^ Cardiology Department of Çorlu State Hospital Tekirdağ Turkiye; ^3^ Department of Cardiology, Sultan II. Abdulhamid Training and Research Hospital University of Health Sciences Istanbul Turkiye; ^4^ Gülhane Health Sciences Institute University of Health Sciences Ankara Turkiye; ^5^ Department of Biochemistry, Hamidiye Faculty of Medicine University of Health Sciences Istanbul Turkiye; ^6^ Department of Histology and Embryology, Hamidiye International Faculty of Medicine University of Health Sciences Istanbul Turkiye

**Keywords:** alcohol‐induced liver injury, alcohol‐related cardiac damage, atorvastatin, hepatoprotection, oxidative stress, rat

## Abstract

Excessive alcohol consumption causes significant cardio‐hepatic damage. This study investigated the effects of standard (10 mg/kg) and high (40 mg/kg) doses of atorvastatin on fibrosis, hypertrophy, apoptosis, inflammation, oxidative stress, mitochondrial‐mediated apoptotic pathways, and thiol/disulfide balance in alcohol‐induced heart and liver injury. Forty Sprague‐Dawley rats were divided into five groups (*n* = 8; 4 male/4 female): Control (CONT), ALCOHOL (2.5 g/kg 20% ethanol i.p./28 days), ATOR (10 mg/kg o.g./28 days), ALCOHOL + ATOR (10 mg/kg o.g./28 days) and ALCOHOL+ hATOR (40 mg/kg o.g./28 days). Histopathologically, alcohol caused cardiac myocyte dilation, vacuolization, inflammation, and hepatic cord disruption. While standard ATOR partially improved tissue architecture (*p* < 0.001), the ALCOHOL+ hATOR group showed the lowest severity. Alcohol exposure significantly increased apoptosis (Caspase‐3, M30, M65), oxidative stress (TOS, OSI, MDA), ischemia (IMA), and inflammatory cytokines (IL‐1β, TNF‐α, HsCRP, Endothelin‐1) across serum, heart, and liver samples, while decreasing TAS, total thiol, and native thiol levels (*p* < 0.001 for all vs. CONT). Conversely, high‐dose atorvastatin (ALCOHOL + hATOR) significantly reversed alcohol‐induced toxicity by decreasing Caspase‐3, M30, M65, TOS, OSI, MDA, TNF‐α, HsCRP, Endothelin‐1, and disulfide levels, while significantly restoring TAS, total thiol, and native thiol levels (*p* < 0.001 vs. ALCOHOL). TAS was significantly higher in the ALCOHOL+hATOR group than the ALCOHOL + ATOR group (*p* < 0.001). Despite marked improvements by hATOR, OSI, TOS, IL‐1β, and HsCRP levels remained higher than CONT (*p* < 0.001). High‐dose atorvastatin (40 mg/kg) exerts robust pleiotropic, anti‐apoptotic, and antioxidant cytoprotection. By preserving thiol‐disulfide homeostasis and limiting inflammation, high‐dose atorvastatin effectively mitigates alcohol‐induced subchronic cardiac and hepatic injury.

## Graphical abstract

Atorvastatin (40 mg/kg) inhibited fibrosis, hypertrophy, apoptosis, inflammation, and oxidative stress caused by alcohol consumption. It may contribute to the regulation of mitochondrial function and thiol/disulfide balance by limiting alcohol‐related heart and liver damage.

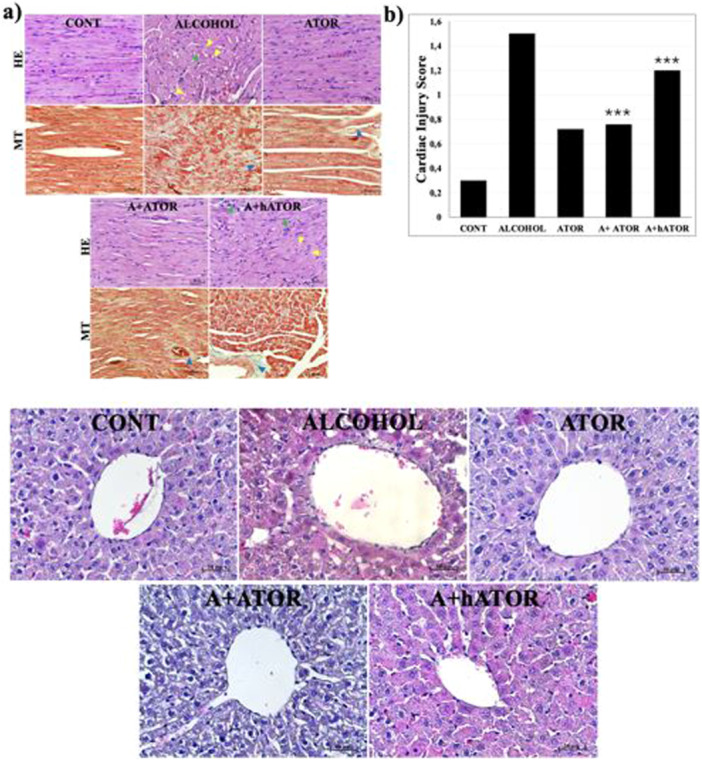

## 1 Introduction

Forty‐three percent of the population aged 15 and over consumes alcohol worldwide [1]. Alcohol consumption is one of the most important factors increasing the risk of morbidity and mortality worldwide [2]. Excessive alcohol consumption causes significant damage to the liver and cardiovascular system organs. It can also cause neoplastic lesions in organs such as the esophagus and breast [3].

Chronic alcohol use causes an increase in cardiovascular diseases worldwide, which is a major financial burden for health systems. Alcohol abuse weakens cardiomyocytes. Ethanol has high reactivity and diffusion rate and can easily cross membranes. In this way, it affects the functions of mitochondria, plasma membrane, endoplasmic reticulum, ribosomes and contractile proteins of cardiomyocytes. It impairs cardiac contraction and mitochondrial oxidative phosphorylation. Alcohol reduces heart contraction, disrupts signaling processes and activates apoptosis. Alcohol reduces cell regeneration and proliferation. Therefore, it also suppresses the repair mechanisms of cardiomyocytes. With these effects, it causes myocardial fibrosis and ventricular dysfunction. Alcohol triggers inflammation and increases cytokines such as tumor necrosis factor‐α (TNF‐α). In chronic alcoholism, the increase in TNF‐α levels is indicative of cardiac pathologies, fibrosis, and necrosis. In addition, ethanol metabolites (acetaldehyde and acetate) can have a direct toxic effect on myocytes. Pathological mechanisms responsible for alcoholic cardiomyopathy may include protein‐aldehyde products, modifications of lipoprotein and apolipoprotein particles, accumulation of fatty acid ethyl esters, and oxidative stress. Due to all these processes, left ventricular systolic function decreases, and left ventricular diameter and mass index increase [4, 5]. The damage caused by alcohol to cardiomyocytes is observed histologically under a light microscope as interstitial fibrosis, myocyte and nuclear hypertrophy, and myocyte necrosis, while under an electron microscope it is seen as fragmentation of contractile elements, edema of the sarcoplasmic reticulum, enlargement of intercalated disks, and fat deposits [4].

Concurrently, the liver is the primary site of ethanol metabolism and a major target for alcohol‐induced toxicity. Chronic alcohol consumption triggers alcoholic liver disease (ALD) through complex, interconnected pathophysiological pathways. The metabolic oxidation of ethanol by alcohol dehydrogenase and cytochrome P450 2E1 (CYP2E1) generates excessive reactive oxygen species (ROS) and highly reactive acetaldehyde, driving severe oxidative stress, lipid peroxidation, and mitochondrial dysfunction within hepatocytes [6]. Furthermore, alcohol abuse disrupts gut barrier integrity, leading to the translocation of lipopolysaccharides into the portal circulation. This activates Kupffer cells via Toll‐like receptor 4 (TLR4) signaling, prompting a cascade of pro‐inflammatory cytokines such as TNF‐α and IL‐1β, which ultimately accelerate hepatic inflammation, steatosis, and progressive fibrosis [6, 7].

Statins are among the drugs frequently used today to protect against cardiovascular diseases. In the early 1970s, Dr. Akira Endo first discovered metabolites that inhibit 3‐hydroxy‐3‐methylglutaril‐coenzyme A (HMG‐CoA) reductase, an important regulatory enzyme in cholesterol biosynthesis in fungi and molds [8]. The success of statins was first demonstrated in patients with familial hypercholesterolemia. The 1994 Scandinavian Simvastatin Survival Study, one of the largest‐scale, randomized statin trials, demonstrated that simvastatin treatment resulted in a 25% reduction in total plasma cholesterol and a 35% reduction in low‐density lipoprotein (LDL) in patients with known coronary heart disease or a history of myocardial infarction [9]. In addition to lowering lipid levels, statins inhibit HMG‐CoA reductase and exhibit beneficial effects in cardiovascular disease. Their pleiotropic effects include inhibition of the isoprenoid pathway leading to activation of endothelial nitric oxide synthase through inhibition of small guanosine triphosphatases (GTPases) from the Rho family, and attenuation of reactive oxygen species through Rac1 inhibition [10, 11]. The pleiotropic effects of statins are dose dependent. High‐dose statins exert an anti‐inflammatory effect by lowering High‐Sensitivity C‐Reactive Protein (Hs‐CRP) and myeloperoxidase levels [12, 13]. Based on previous dose‐response and translational studies in rodent models, a standard dose of 10 mg/kg atorvastatin primarily regulates lipid profiles, whereas a high dose of 40 mg/kg has been safely utilized to maximize cholesterol‐independent pleiotropic, antioxidant, and tissue‐protective pathways without inducing overt toxicity [13]. Thus, evaluating both 10 and 40 mg/kg provides a justified translational rationale to observe dose‐dependent protective mechanisms.

In a study modeling alcohol‐related cardiac damage in mice, simvastatin was observed to significantly reduce alcohol‐related cardiomyocyte hypertrophy, cardiac fibrosis, and inflammation [5]. In another study, high‐dose atorvastatin was shown to alleviate ischemia/reperfusion injury after kidney transplantation in rats by reducing oxidative stress, reactive oxygen species, and inflammation [13]. The effect of 6‐gingerol against alcohol‐related reactive oxygen species‐mediated cardiac tissue damage in rats was investigated, and it was found that 6‐gingerol suppressed the production of reactive oxygen species in cardiac tissue [14].

A review of the literature reveals that, in addition to their plasma lipid‐lowering effects, statins are utilized in various fields due to their antioxidant and anti‐inflammatory effects. While there are limited studies on atorvastatin's effects on endoplasmic reticulum stress and lipid metabolism regulation in relation to alcohol toxicity, there is no comprehensive study investigating atorvastatin's effects in preventing alcohol‐related cardiac damage. Our study is unique in this respect. We hypothesized that atorvastatin would attenuate alcohol‐induced cardio‐hepatic damage in a dose‐dependent manner by suppressing oxidative stress, inflammation, and apoptosis, while preserving thiol‐disulfide homeostasis. Therefore, this study investigated the effects of atorvastatin on fibrosis, hypertrophy, apoptosis, inflammation, oxidative stress, mitochondrial function, and thiol/disulfide balance regulation in alcohol‐induced heart and liver injury.

## 2 Materials and Methods

The animals used in the study were 10–12 weeks old and weighed an average of 200 ± 30 g. Twenty male and 20 female Sprague‐Dawley rats were used. The rats were provided with food and water continuously. The ambient temperature was 22°C ± 4°C, humidity was 65%–70%, and a 12‐h light‐dark period was applied. The study was approved by the Hamidiye Animal Experiments Local Ethics Committee of Health Sciences University (approval date: January 15, 2025; approval number: 01/02).

The animals were divided into five groups (*n*:8) using a simple randomization method. Each group contained four male and four female rats. Sample size was calculated using GPower 3.1 software. In this study, 90% power, a 95% confidence interval, and a sample effect size of *d* = 0.5 were calculated. The minimum sample size was calculated as 8 animals for each group, and due to ethical constraints, 40 animals were included. The experimental groups (ALCOHOL, ALCOHOL + ATOR, and ALCOHOL + hATOR) were administered 20% v/v ethyl alcohol at a dose of 2.5 g/kg once daily intraperitoneally (i.p.) for 28 days. To evaluate a co‐treatment paradigm, atorvastatin or saline was administered orally 2 h after the ethanol injection each day. Atorvastatin (ATOR, ALCOHOL + ATOR, and ALCOHOL + hATOR) and saline solution (CONT and ALCOHOL groups) were administered orally (o.g.). The volume for oral gavage was adjusted to 1 ml per animal. Based on previous studies, the standard dose of atorvastatin has been determined as 10 mg/kg, with a high dose of 40 mg/kg [13]. In this study, a commercially available preparation of atorvastatin (LIPITOR 20 mg, Viatris Ilaclari Limited Sirketi, Turkiye) was used. The tablet was thoroughly crushed in a mortar, dissolved in physiological saline (0.9% NaCl, which served as the vehicle), filtered, and then used immediately after preparation.

The 28‐day, 2.5 g/kg ip ethanol daily protocol applied in our study is a subchronic/chronic toxicity model accepted in the literature to mimic chronic alcoholic cardiomyopathy and associated myocardial damage in rats. Considering the rat life cycle, this 4‐week period corresponds to long‐term and continuous alcohol exposure in humans. This model provides sufficient duration and dose to trigger permanent mitochondrial dysfunction, calcium homeostasis disturbances, chronic oxidative stress due to “Cytochrome P450 family 2 subfamily E member 1” (CYP2E1) activation, and structural myocardial remodeling (fibrosis) accompanied by cardiomyocyte apoptosis, unlike the transient effects of acute alcohol consumption.

Following an overnight (12‐h) fasting period on the last day of the experiment, sacrifice was performed under general anesthesia (80 mg/kg ketamine and 10 mg/kg xylazine i.p.) via cervical dislocation. Heart and liver tissue samples, as well as intracardiac blood samples, were taken from the animals. Researchers conducting histological and biochemical analyses on blood and tissue samples taken from animals had no prior knowledge of the animal groups they were examining. Histopathological examinations and semi‐quantitative scoring were performed independently by two senior pathologists blinded to the study groups. The inter‐observer reliability was assessed using Cohen's kappa coefficient (*κ*), demonstrating an excellent agreement between the two observers (*κ* = 0.88).

### 2.1 Experimental Groups

– **CONT (*n*** = **8)**: Physiological saline (1 mL, o.g./28 days).
– **ALCOHOL (*n*** = **8)**: 2.5 g/kg 20% alcohol (i.p./28 days) + physiological saline (1 mL, o.g./28 days).
– **ATOR (*n*** = **8)**: Atorvastatin 10 mg/kg (o.g./28 days).
– **ALCOHOL** + **ATOR (*n*** = **8)**: 2.5 g/kg 20% alcohol (i.p./28 days) + Atorvastatin 10 mg/kg (o.g./28 days).
– **ALCOHOL** + **hATOR (*n*** = **8)**: 2.5 g/kg 20% alcohol (i.p./28 days) + Atorvastatin 40 mg/kg (o.g./28 days).

*Sample Collection and Storage*: The portion of the heart containing the ventricles and representative liver tissue samples were preserved in 10% neutral‐buffered formalin (Sigma‐Aldrich, USA; HT501128 Formalin solution) for histopathological examination. The remaining heart and liver tissue samples were placed in sterile polypropylene microcentrifuge tubes (Isolab, Turkiye) for biochemical analysis and stored at −80°C until analysis. Before biochemical measurements, tissue samples were homogenized in 1× phosphate‐buffered saline/dPBS (0.1 mol/L, pH 7.4; Thermo Scientific, USA) using ceramic homogenization beads (MPBio, USA) and a tissue homogenizer (MPBio, USA). The homogenates were centrifuged at 10,000 × *g* for 10 min at +4°C using a refrigerated centrifuge (Nuve, Turkiye), and the resulting supernatants were used for biochemical analyses. The selection of both cardiac and hepatic tissues for investigating oxidative damage and protection mechanisms aligns with established experimental protocols in recent literature [15, 16]. Total protein concentration in tissue homogenates was determined by the Bradford protein assay according to the method of Bradford [17], and tissue biomarker results were normalized to total protein content. Protein concentration was used only for technical normalization of tissue homogenate results and was not evaluated as an independent outcome variable. Intracardiac blood samples were collected into sterile gel‐containing biochemistry tubes (Vacusera, Turkiye). After centrifugation at 3000 × *g* for 10 min, serum samples were aliquoted and stored at −80°C until analysis.

### 2.2 Biochemical Assessments

*Determination of Biochemical Parameters in Serum and Tissue*: Total antioxidant level (TAS), total oxidant level (TOS), total thiol, native thiol, and ischemia‐modified albumin (IMA) were measured using commercially available photometric/colorimetric assay kits according to the manufacturers' instructions. TAS was measured using a Total Antioxidant Status Assay Kit (Rel Assay Diagnostics, Gaziantep, Türkiye; Cat. No. RL0017), based on the automated colorimetric method described by Erel [18]. TOS was measured using a Total Oxidant Status Assay Kit (Rel Assay Diagnostics, Gaziantep, Türkiye; Cat. No. RL0024), based on the automated colorimetric method described by Erel [19]. Total thiol and native thiol levels were determined using Total Thiol and Native Thiol Assay Kits (Rel Assay Diagnostics, Gaziantep, Türkiye; Cat. No. RL0192 and RL0185, respectively), according to the automated thiol/disulfide homeostasis method described by Erel and Neselioglu [20]. Disulfide level was calculated as (total thiol − native thiol)/2. Oxidative stress index (OSI) was calculated as the ratio of TOS to TAS. IMA was measured using an Ischemia‐Modified Albumin Assay Kit, and expressed as absorbance units [21]. Tissue results were normalized to total protein concentration determined by the Bradford method.

*ELISA Studies and Analytical Quality Control*: Serum and tissue homogenate levels of High‐Sensitivity C‐Reactive Protein (HsCRP), interleukin‐1‐beta (IL‐1β), TNF‐α, Endothelin‐1, Caspase‐3, Cytokeratin 18‐M30, Cytokeratin 18‐M65, and malondialdehyde (MDA) were measured using commercially available ELISA kits according to the manufacturers' protocols. The following kits were used: rat HsCRP ELISA Kit (Elabscience Biotechnology Inc., Wuhan, China; Cat. No. E‐EL‐R3002; pg/mL), rat IL‐1β ELISA Kit (Elabscience Biotechnology Inc.; Cat. No. E‐EL‐R0012; pg/mL), rat TNF‐α ELISA Kit (Elabscience Biotechnology Inc.; Cat. No. E‐EL‐R2856; pg/mL), rat Endothelin‐1 ELISA Kit (Elabscience Biotechnology Inc.; Cat. No. E‐EL‐R1458; pg/mL), rat Caspase‐3 ELISA Kit (Elabscience Biotechnology Inc.; Cat. No. E‐EL‐R0160; ng/mL), rat Cytokeratin 18‐M30 ELISA Kit (MyBioSource, San Diego, CA, USA; Cat. No. MBS9141527; ng/mL), rat Cytokeratin 18‐M65 ELISA Kit (MyBioSource; Cat. No. MBS1607541; ng/L), and MDA ELISA Kit (Elabscience Biotechnology Inc.; Cat. No. E‐EL‐0060; ng/mL). These biochemical markers, evaluating oxidative stress, inflammatory response, and apoptotic pathways in rat tissue homogenates, were selected based on validated toxicological and tissue‐protective models described previously [15, 16]. Absorbance was measured using a microplate reader (BioTek Synergy, HTX Multimode Reader, USA) at the wavelength recommended by each manufacturer. All standards, controls, serum samples, and tissue homogenates were analyzed in duplicate, and the mean of duplicate measurements was used for statistical analysis. Duplicate measurements with a coefficient of variation greater than 10% were repeated. Manufacturer‐provided validation characteristics, including analytical sensitivity, detection range, specificity/cross‐reactivity statements, and intra‐/inter‐assay variability where available, were considered during assay validation. Tissue homogenate results were normalized to total protein content determined by the Bradford method and expressed per mg protein where applicable.

### 2.3 Histopathological Examination of Cardiac and Liver Tissue

Paraffin‐embedded heart and liver tissues were cut into 5 μm‐thick sections, followed by deparaffinization in sequential steps of xylene and ethanol solutions (100%, 96%, and 70%; Merck, Darmstadt, Germany), and a final rehydration step in distilled water. For histological analysis, H&E staining was performed to evaluate overall tissue morphology, while Masson's Trichrome staining was applied to visualize collagen distribution among the groups using a commercial kit (Bio‐Optica, Milan, Italy; Masson Trichrome Kit, Cat No: 04‐010802). Histological images were captured using an Axio Imager.A1 microscope (Carl Zeiss, Oberkochen, Germany) at 40× magnification.

The degree of heart tissue injury was assessed using semiquantitative scoring on HE stained preparations. In short, histopathological changes were graded on a scale of 0 to 3 depending on the severity of myocyte changes, as shown below:

0: Normal tissue architecture.

1: Localized or scattered leukocytes, with 3–5 vacuolated myocytes.

2: Focally aggregated leukocyte clusters and vacuolation in more than 50% of the myocytes, with a small number of hypertrophic myocytes.

3: Leukocyte clusters occupying more than 50% of the area, accompanied by marked myocyte hypertrophy or focal regions of necrotic cells.

In the liver sections; degree of steatosis was scored from 0 to 3 based on the proportion of hepatocytes affected in the liver sections (0, < 10%; 1, 10%–33%; 2, 33%–66%; and 3, > 66% of hepatocytes) [22].

### 2.4 Statistical Analysis

All statistical analyses were performed using IBM SPSS Statistics software, version 27.0 (IBM Corporation, Armonk, NY, USA). The normality of data distributions was assessed using the Shapiro‐Wilk test. Continuous variables conforming to a normal distribution are expressed as mean ± standard deviation and were analyzed using one‐way analysis of variance (ANOVA), followed by Tukey's Honest Significant Difference (HSD) post hoc test for pairwise comparisons. Additionally, to account for Sex as a Biological Variable (SABV) in line with ARRIVE 2.0 guidelines, a two‐factor ANOVA (Two‐Way ANOVA) was performed on normally distributed data to evaluate the main effects of treatment, sex, and their interaction (sex × treatment). Data not satisfying the assumption of normality are presented as median (interquartile range) and were analyzed using the Kruskal‐Wallis test, with Dunn's post hoc correction for multiple comparisons. Statistical significance was set at a two‐tailed *p* value of less than 0.05, and exact *p* values are reported throughout the manuscript except where *p* < 0.001.

## 3 Results

Two‐Way ANOVA and non‐parametric sub‐group analyses revealed a highly significant main effect for the experimental treatments across all evaluated biochemical parameters and histopathological scores (*p* < 0.001). However, no statistically significant main effect was observed for biological sex, and no significant sex × treatment interaction was detected (*p* > 0.05), indicating a uniform response profile between male and female cohorts within our sample framework.

### 3.1 Histopathology of the Heart and Liver

**Figure 1:**
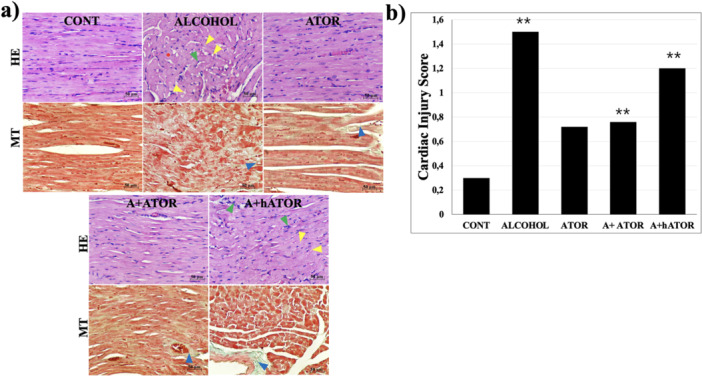
Histopathological evaluation of cardiac tissue. (a) Representative H&E and Masson's Trichrome (MT) staining of cardiac sections. Control (CONT) hearts display normal myocardial architecture with cardiomyocytes containing centrally located nuclei. Alcohol‐treated groups exhibit marked structural disruption characterized by dilated cardiac myocytes with enlarged nuclei (red star), loss of cellular organization, diffuse or focal inflammatory infiltrates composed of neutrophils and/or lymphocytes (green arrowhead), and prominent cytoplasmic vacuolization (yellow arrowhead). Standard dose of ATOR‐treated group (A + ATOR) shows partial morphological restoration, though cytoplasmic vacuolization persists (yellow arrowhead). MT staining demonstrates normal perivascular collagen distribution (blue arrow) across control and experimental groups. Scale bar = 50 µm. (b) Semi‐quantitative scoring of cardiac tissue alterations. **p < 0.01 compared to CONT group. A: Alcohol; ATOR: Atorvastatin; CONT: Control; hATOR: High‐dose Atorvastatin.

Compared to the CONT group, alcohol exposure led to a noticeable deterioration of cardiac tissue organization: myocytes appeared dilated with enlarged nuclei, inflammatory cells accumulated either focally or diffusely within the tissue, and many fibers displayed cytoplasmic vacuolization. Animals receiving the standard dose of ATOR after alcohol exposure showed an improvement in overall tissue integrity when compared with the alcohol‐only group, however, some vacuolated areas were still evident (Figure 1a). MT staining showed that the perivascular collagen structure remained largely unaffected in all groups. These observations were consistent with the semi‐quantitative scoring (Figure 1b), which indicated a significant increase in injury scores in the alcohol group and a measurable reduction (*p* < 0.001) following standard‐dose ATOR treatment.

**Figure 2:**
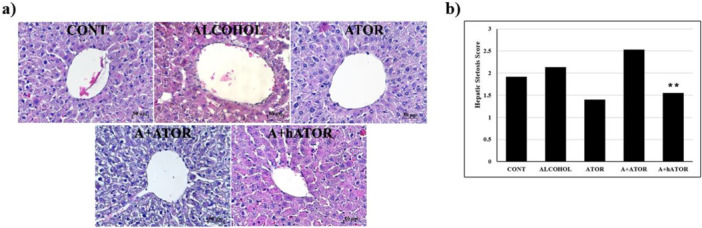
Histology of liver sections. (a) Control (CONT) livers show normal hepatic architecture, whereas alcohol exposure disrupts hepatic cords with increased lipid accumulation, which is noticeably reduced in the hATOR‐treated compared to low dose group. (b) Semi‐quantitative scoring of liver tissue changes. **p < 0.01 compared to CONT group. A: Alcohol; ATOR: Atorvastatin; CONT: Control; hATOR: High‐dose Atorvastatin.

In the liver, the alcohol group showed marked disruption of hepatic cord organization accompanied by cytoplasmic vacuolization within hepatocytes. Treatment with ATOR resulted in a partial improvement in hepatic architecture, with a noticeable reduction in these changes compared to the alcohol group. Notably, liver histology in the A + hATOR group appeared less severe than that observed in the ALCOHOL + ATOR group (Figure 2a).

### 3.2 Apoptotic Process

*Caspase‐3*: In serum, heart, and liver tissue samples, Caspase‐3 levels were found to be significantly increased in the ALCOHOL group compared to all other groups (*p* < 0.001). Following treatment, Caspase‐3 levels were significantly decreased in the ALCOHOL + hATOR group when compared directly with the ALCOHOL‐only group (*p* < 0.001); Tables 1, 2, 3).

**Table 1:** Biochemical parameters of peripheral blood plasma.

Variables	Groups	Mean (±standard deviation)	*p* value	Subgroup comparisons	Effect size (Cohen's *d*)
TOS (µmol H_2_O_2_ Eq./L)	CONT ALCOHOL ATOR ALCOHOL + ATOR ALCOHOL + hATOR	5.05 ± 0.56 18.30 ± 1.12 5.86 ± 0.42 13.75 ± 0.97 8.05 ± 0.45	< 0.001	2 > 1,3,4,5 4 > 1,3,5 5 > 1,3	12.01
TAS (mmol Trolox Eq./L)	CONT ALCOHOL ATOR ALCOHOL + ATOR ALCOHOL + hATOR	1.07 ± 0.02 0.28 ± 0.05 0.94 ± 0.02 0.44 ± 0.05 0.84 ± 0.02	< 0.001	1 > 2,3,4,5 3 > 2,4,5 4 > 2 5 > 2,4	14.71
OSI (AU)	CONT ALCOHOL ATOR ALCOHOL + ATOR ALCOHOL + hATOR	4.73 ± 0.58 67.88 ± 13.72 6.20 ± 0.45 31.58 ± 4.38 9.62 ± 0.54	< 0.001	2 > 1,3,4,5 4 > 1,3,5 5 > 3	6.00
HsCRP (7.81–500 pg/mL)	CONT ALCOHOL ATOR ALCOHOL + ATOR ALCOHOL + hATOR	60.61 ± 0.34 328.92 ± 32.18 108.56 ± 11.21 280.85 ± 24.85 120.92 ± 16.76	< 0.001	2 > 1,3,4,5 3 > 1 4 > 1,3,5 5 > 1	8.11
IL‐1 beta (31.25–2000 pg/mL)	CONT ALCOHOL ATOR ALCOHOL + ATOR ALCOHOL + hATOR	66.21 ± 8.83 770.08 ± 28.48 151.57 ± 7.55 587.27 ± 17.78 210.49 ± 13.15	< 0.001	2 > 1,3,4,5 3 > 1 4 > 1,3,5 5 > 1,3	25.23
TNF‐alpha (15.63–1000 pg/mL)	CONT ALCOHOL ATOR ALCOHOL + ATOR ALCOHOL + hATOR	52.05 ± 1.11 433.71 ± 14.51 82.47 ± 6.83 337.08 ± 25.00 118.35 ± 15.71	< 0.001	2 > 1,3,4,5 3 > 1 4 > 1,3,5 5 > 1,3	20.85
IMA (AbsU)	CONT ALCOHOL ATOR ALCOHOL + ATOR ALCOHOL + hATOR	0.31 ± 0.01 0.70 ± 0.01 0.35 ± 0.02 0.59 ± 0.02 0.39 ± 0.01	< 0.001	2 > 1,3,4,5 3 > 1 4 > 1,3,5 5 > 1,3	31.00
Endothelin‐1 (0.78–50 pg/mL)	CONT ALCOHOL ATOR ALCOHOL + ATOR ALCOHOL + hATOR	1.35 ± 0.23 18.07 ± 0.53 5.17 ± 0.46 14.67 ± 0.66 6.20 ± 2.66	< 0.001	2 > 1,3,4,5 3 > 1 4 > 1,3,5 5 > 1	6.19
Caspase‐3 (0.31–20 ng/mL)	CONT ALCOHOL ATOR ALCOHOL + ATOR ALCOHOL + hATOR	0.50 ± 0.20 5.64 ± 0.43 1.23 ± 0.25 4.50 ± 0.60 1.79 ± 0.67	< 0.001	2 > 1,3,4,5 4 > 1,3,5 5 > 1	6.84
Cytokeratin 18‐M30 (0.1–25 ng/mL)	CONT ALCOHOL ATOR ALCOHOL + ATOR ALCOHOL + hATOR	0.28 ± 0.07 7.00 ± 0.16 1.03 ± 0.07 5.53 ± 0.27 1.47 ± 0.34	< 0.001	2 > 1,3,4,5 3 > 1 4 > 1,3,5 5 > 1,3	20.81
Cytokeratin 18‐M65 (15–3000 ng/L)	CONT ALCOHOL ATOR ALCOHOL + ATOR ALCOHOL + hATOR	17.40 ± 2.21 95.91 ± 2.49 23.84 ± 2.49 66.56 ± 1.38 25.57 ± 3.11	< 0.001	2 > 1,3,4,5 3 > 1 4 > 1,3,5 5 > 1	24.97
MDA (31.25–2000 ng/mL)	CONT ALCOHOL ATOR ALCOHOL + ATOR ALCOHOL + hATOR	40.67 ± 2.27 220.53 ± 1.46 68.72 ± 4.76 165.98 ± 10.69 77.90 ± 7.91	< 0.001	2 > 1,3,4,5 3 > 1 4 > 1,3,5 5 > 1	25.08
Total thiol (µmol/L)	CONT ALCOHOL ATOR ALCOHOL + ATOR ALCOHOL + hATOR	502.03 ± 44.97 279.45 ± 30.53 457.13 ± 24.33 332.93 ± 12.37 405.57 ± 22.43	< 0.001	1 > 2,4,5 3 > 2,4 4 > 2 5 > 2,4	4.71
Native thiol (µmol/L)	CONT ALCOHOL ATOR ALCOHOL + ATOR ALCOHOL + hATOR	442.14 ± 28.88 4.01 ± 0.91 258.63 ± 9.50 95.38 ± 3.44 236.63 ± 6.93	< 0.001	1 > 2,3,4,5 3 > 2,4,5 4 > 2 5 > 2,4	47.07
Disulfide (µmol/L)	CONT ALCOHOL ATOR ALCOHOL + ATOR ALCOHOL + hATOR	29.94 ± 12.45 137.72 ± 15.36 99.24 ± 13.36 118.77 ± 5.91 84.47 ± 12.22	< 0.001	2 > 1,3,5 3 > 1 4 > 1,5 5 > 1	3.84

*Note:* Cohen's *d* effect size was calculated to quantify the magnitude of the therapeutic response between the disease control (ALCOHOL group) and the high‐dose treatment (ALCOHOL + hATOR group). According to Cohen's statistical criteria, *d* > 0.8 denotes a remarkably large and robust experimental effect size.

*Note* for Subgroup Comparisons: All explicitly marked post‐hoc pairwise discrepancies (e.g., 2 > 1) indicate robust statistical significance established at the *p* < 0.01 or *p* < 0.001 threshold based on Bonferroni/Tukey multi‐comparison adjustment.

Group Definitions: 1: CONT, 2: ALCOHOL, 3: ATOR, 4: ALCOHOL + ATOR, 5: ALCOHOL + hATOR.

**Table 2:** Biochemical parameters of heart tissue.

Variables	Groups	Mean (±standard deviation)	*p* value	Subgroup comparisons	Effect size (Cohen's *d*)
TOS (µmol H_2_O_2_ Eq./L mg protein)	CONT ALCOHOL ATOR ALCOHOL + ATOR ALCOHOL + hATOR	8.75 ± 0.58 18.82 ± 0.15 9.35 ± 0.42 16.18 ± 0.35 10.66 ± 0.47	< 0.001	2 > 1,3,4,5 4 > 1,3,5 5 > 1,3	23.39
TAS (mmol Trolox Eq./L mg protein)	CONT ALCOHOL ATOR ALCOHOL + ATOR ALCOHOL + hATOR	1.19 ± 0.01 0.42 ± 0.02 1.00 ± 0.01 0.65 ± 0.02 0.97 ± 0.01	< 0.001	1 > 2,3,4,5 3 > 2,4 4 > 2 5 > 2,4	34.79
OSI (AU)	CONT ALCOHOL ATOR ALCOHOL + ATOR ALCOHOL + hATOR	7.31 ± 0.49 45.08 ± 2.07 9.39 ± 0.47 24.85 ± 1.20 10.95 ± 0.54	< 0.001	2 > 1,3,4,5 3 > 1 4 > 1,3,5 5 > 1	22.56
IL‐1 beta (31.25–2000 pg/mL mg protein)	CONT ALCOHOL ATOR ALCOHOL + ATOR ALCOHOL + hATOR	177.56 ± 13.48 1080.03 ± 52.92 309.17 ± 30.79 801.76 ± 42.26 335.62 ± 66.03	< 0.001	2 > 1,3,4,5 3 > 1 4 > 1,3,5 5 > 1	12.44
TNF‐alpha (15.63–1000 pg/mL mg protein)	CONT ALCOHOL ATOR ALCOHOL + ATOR ALCOHOL + hATOR	306.84 ± 35.49 640.27 ± 73.55 358.20 ± 24.68 563.87 ± 19.96 390.65 ± 15.51	< 0.001	2 > 1,3,4,5 4 > 1,3,5 5 > 1	4.70
Endothelin‐1 (0.78–50 pg/mL mg protein)	CONT ALCOHOL ATOR ALCOHOL + ATOR ALCOHOL + hATOR	5.40 ± 2.37 27.50 ± 2.27 8.70 ± 1.93 20.07 ± 3.94 10.15 ± 2.71	< 0.001	2 > 1,3,4,5 4 > 1,3,5 5 > 1	6.94
Caspase‐3 (0.31–20 ng/mL mg protein)	CONT ALCOHOL ATOR ALCOHOL + ATOR ALCOHOL + hATOR	3.51 ± 0.56 10.79 ± 0.14 4.08 ± 0.97 8.52 ± 1.25 5.14 ± 0.93	< 0.001	2 > 1,3,4,5 4 > 1,3,5 5 > 1	8.50
Cytokeratin 18‐M30 (0.1–25 ng/mL mg protein)	CONT ALCOHOL ATOR ALCOHOL + ATOR ALCOHOL + hATOR	2.11 ± 0.38 8.74 ± 0.50 2.91 ± 0.12 6.79 ± 0.34 3.68 ± 0.29	< 0.001	2 > 1,3,4,5 3 > 1 4 > 1,3,5 5 > 1,3	12.38
Cytokeratin 18‐M65 (15–3000 ng/L mg protein)	CONT ALCOHOL ATOR ALCOHOL + ATOR ALCOHOL + hATOR	113.39 ± 4.80 291.95 ± 6.03 134.15 ± 5.00 238.50 ± 4.35 152.30 ± 6.48	< 0.001	2 > 1,3,4,5 3 > 1 4 > 1,3,5 5 > 1,3	22.31
MDA (31.25–2000 ng/mL mg protein)	CONT ALCOHOL ATOR ALCOHOL + ATOR ALCOHOL + hATOR	111.55 ± 6.58 457.56 ± 11.51 141.72 ± 5.82 352.58 ± 14.38 179.87 ± 11.00	< 0.001	2 > 1,3,4,5 3 > 1 4 > 1,3,5 5 > 1,3	24.67

*Note:* Cohen's d effect size was calculated to quantify the magnitude of the therapeutic response between the disease control (ALCOHOL group) and the high‐dose treatment (ALCOHOL + hATOR group). According to Cohen's statistical criteria, *d* > 0.8 denotes a remarkably large and robust experimental effect size.

*Note* for Subgroup Comparisons: All explicitly marked post‐hoc pairwise discrepancies (e.g., 2 > 1) indicate robust statistical significance established at the *p* < 0.01 or *p* < 0.001 threshold based on Bonferroni/Tukey multi‐comparison adjustment.

Group Definitions: 1: CONT, 2: ALCOHOL, 3: ATOR, 4: ALCOHOL + ATOR, 5: ALCOHOL + hATOR.

**Table 3:** Biochemical parameters of liver tissue.

Variables	Groups	Mean (±standard deviation)	*p* value	Subgroup comparisons	Effect size (Cohen's *d*)
TOS (µmol H_2_O_2_ Eq./L mg protein)	CONT ALCOHOL ATOR ALCOHOL + ATOR ALCOHOL + hATOR	8.46 ± 0.83 18.56 ± 0.30 10.68 ± 0.14 16.64 ± 0.14 11.75 ± 0.17	< 0.001	2 > 1,3,4,5 3 > 1 4 > 1,3,5 5 > 1,3	27.85
TAS (mmol Trolox Eq./L mg protein)	CONT ALCOHOL ATOR ALCOHOL + ATOR ALCOHOL + hATOR	1.20 ± 0.01 0.42 ± 0.02 1.05 ± 0.02 0.64 ± 0.02 0.95 ± 0.02	< 0.001	1 > 2,3,4,5 3 > 2,4,5 4 > 2 5 > 2,4	26.51
OSI (AU)	CONT ALCOHOL ATOR ALCOHOL + ATOR ALCOHOL + hATOR	7.08 ± 0.70 43.94 ± 2.35 10.20 ± 0.30 26.22 ± 0.90 12.38 ± 0.31	< 0.001	2 > 1,3,4,5 3 > 1 4 > 1,3,5 5 > 1,3	18.81
IL‐1 beta (31.25–2000 pg/mL mg protein)	CONT ALCOHOL ATOR ALCOHOL + ATOR ALCOHOL + hATOR	364.75 ± 27.38 1034.77 ± 39.40 515.91 ± 30.08 943.52 ± 17.03 601.35 ± 22.60	< 0.001	2 > 1,3,4,5 3 > 1 4 > 1,3,5 5 > 1,3	13.51
TNF‐alpha (15.63–1000 pg/mL mg protein)	CONT ALCOHOL ATOR ALCOHOL + ATOR ALCOHOL + hATOR	441.20 ± 19.60 690.70 ± 46.33 489.44 ± 10.70 634.35 ± 10.77 527.71 ± 14.43	< 0.001	2 > 1,3,4,5 3 > 1 4 > 1,3,5 5 > 1	4.74
Endothelin‐1 (0.78–50 pg/mL mg protein)	CONT ALCOHOL ATOR ALCOHOL + ATOR ALCOHOL + hATOR	6.71 ± 1.88 29.32 ± 2.91 8.94 ± 1.24 24.08 ± 1.90 10.54 ± 1.45	< 0.001	2 > 1,3,4,5 4 > 1,3,5 5 > 1	8.20
Caspase‐3 (0.31–20 ng/mL mg protein)	CONT ALCOHOL ATOR ALCOHOL + ATOR ALCOHOL + hATOR	3.51 ± 0.33 10.24 ± 0.30 4.71 ± 1.00 7.68 ± 1.15 4.85 ± 0.43	< 0.001	2 > 1,3,4,5 3 > 1 4 > 1,3,5 5 > 1	14.54
Cytokeratin 18‐M30 (0.1–25 ng/mL mg protein)	CONT ALCOHOL ATOR ALCOHOL + ATOR ALCOHOL + hATOR	2.11 ± 0.38 8.74 ± 0.50 2.91 ± 0.12 6.79 ± 0.34 3.68 ± 0.29	< 0.001	2 > 1,3,4,5 3 > 1 4 > 1,3,5 5 > 1,3	12.38
Cytokeratin 18‐M65 (15–3000 ng/L mg protein)	CONT ALCOHOL ATOR ALCOHOL + ATOR ALCOHOL + hATOR	117.06 ± 5.02 292.71 ± 6.26 140.63 ± 3.74 245.52 ± 2.03 152.44 ± 4.86	< 0.001	2 > 1,3,4,5 3 > 1 4 > 1,3,5 5 > 1,3	25.04
MDA (31.25–2000 ng/mL mg protein)	CONT ALCOHOL ATOR ALCOHOL + ATOR ALCOHOL + hATOR	116.30 ± 4.63 481.55 ± 11.18 138.84 ± 8.57 351.97 ± 12.87 179.55 ± 9.12	< 0.001	2 > 1,3,4,5 3 > 1 4 > 1,3,5 5 > 1,3	29.63

*Note:* Cohen's d effect size was calculated to quantify the magnitude of the therapeutic response between the disease control (ALCOHOL group) and the high‐dose treatment (ALCOHOL + hATOR group). According to Cohen's statistical criteria, *d* > 0.8 denotes a remarkably large and robust experimental effect size.

*Note* for Subgroup Comparisons: All explicitly marked post‐hoc pairwise discrepancies (e.g., 2 > 1) indicate robust statistical significance established at the *p* < 0.01 or *p* < 0.001 threshold based on Bonferroni/Tukey multi‐comparison adjustment.

Group Definitions: 1: CONT, 2: ALCOHOL, 3: ATOR, 4: ALCOHOL + ATOR, 5: ALCOHOL + hATOR.

*Cytokeratin 18‐M30*: Evaluation of serum and tissue homogenates showed that Cytokeratin 18‐M30 levels sharply rose in response to alcohol exposure, marking a significant increase compared to the CONT group (*p* < 0.001). Conversely, high‐dose atorvastatin administration (ALCOHOL + hATOR) successfully suppressed M30 levels in all examined samples relative to the ALCOHOL group (*p* < 0.001); Tables 1, 2, 3).

*Cytokeratin 18‐M65*: Similar to the M30 trends, Cytokeratin 18‐M65 levels in serum, heart, and liver tissues were highest in the ALCOHOL group, showing a statistically significant difference against the control cohorts (*p* < 0.001). Treatment with high‐dose atorvastatin led to a measurable and significant reduction in M65 expression compared to the non‐treated alcohol group (*p* < 0.001); Tables 1, 2, 3).

### 3.3 Oxidative Stress and Antioxidant Capacity

*Total Antioxidant Level*: In serum, heart, and liver tissue samples, TAS was found to be significantly decreased in the ALCOHOL group (*p* < 0.001). When the ATOR, ALCOHOL + ATOR, and ALCOHOL + hATOR groups were compared with the ALCOHOL group, TAS levels were found to be significantly higher in these groups (*p* < 0.001). Furthermore, TAS was found to be higher in the ALCOHOL + hATOR group compared to the ALCOHOL + ATOR group (*p* < 0.001; Tables 1, 2, 3).

*Total Oxidant Level*: In all samples, TOS was found to be significantly increased in the ALCOHOL group compared to the other groups (*p* < 0.001). When the ALCOHOL + hATOR group was compared with the CONT group, the difference between them was found to be significant and high (*p* < 0.001; Tables 1, 2, 3).

*Oxidative Stress Index*: In all samples examined, the OSI level was found to be significantly higher in the ALCOHOL group compared to other groups (*p* < 0.001). In the ALCOHOL + ATOR group, the OSI level was higher compared to the CONT, ATOR, and ALCOHOL + hATOR groups. Although the OSI value was significantly reduced in the ALCOHOL+hATOR group, it was still higher compared to the CONT group (*p* < 0.001; Tables 1, 2, 3).

*MDA Value*: The MDA value obtained from all tissue samples examined was found to be significantly higher in the ALCOHOL group compared to other groups (*p* < 0.001; Tables 1, 2, 3).

### 3.4 Inflammatory Cytokine Response and Anti‐Inflammatory Response

*Interleukin‐1 Beta*: In serum and tissue samples, it was noted that Interleukin‐1 Beta levels were significantly increased in the ALCOHOL group compared to other groups (*p* < 0.001). In the ALCOHOL + hATOR group, IL‐1β levels were found to be higher compared to the CONT and ATOR groups (Tables 1, 2, 3).

*Tumor Necrosis Factor Alpha*: When TNF‐α levels were examined in all samples, it was determined that they were significantly increased in the ALCOHOL group (*p* < 0.001), while they were lower in the ALCOHOL + hATOR group compared to the ALCOHOL group (*p* < 0.001). Although the liver TNF‐α level in the ALCOHOL + hATOR group (527.71 ± 14.43 pg/mL) was numerically higher than that of the ATOR alone group (489.44 ± 10.70 pg/mL), this difference was not statistically significant (*p* > 0.05; Tables 1, 2, 3).

*High‐Sensitivity C‐Reactive Protein*: Serum HsCRP levels were found to be lowest in the CONT group and highest in the ALCOHOL group. In the ALCOHOL + hATOR group, this value was higher compared to the CONT group but significantly decreased (*p* < 0.001; Table 1).

*Endothelin‐1*: In all samples examined, Endothelin‐1 levels were observed to be higher in the ALCOHOL group compared to all other groups and decreased in the ALCOHOL + hATOR group (*p* < 0.001; Tables 1, 2, 3).

### 3.5 Ischemia Status

*Ischemia Modified Albumin*: Serum IMA levels were found to be significantly higher in the ALCOHOL group compared to the CONT group (*p* < 0.001), and significantly lower in the ALCOHOL + hATOR group (*p* < 0.001; Table 1).

### 3.6 Thiol‐Disulfide Homeostasis

*Total Thiol*: Serum thiol levels were found to be lower in the ALCOHOL group compared to all other groups and increased in the ALCOHOL + ATOR and ALCOHOL + hATOR groups (*p* < 0.001; Table 1).

*Native Thiol*: Serum native thiol levels were found to be significantly lower in the ALCOHOL group compared to the CONT group but increased in the ALCOHOL + hATOR group compared to the ALCOHOL group (*p* < 0.001; Table 1).

*Disulfide*: Serum disulfide levels were found to be higher in the ALCOHOL group compared to all other groups. When the ALCOHOL + hATOR group was compared with the ALCOHOL group, a decrease in serum disulfide levels was observed (*p* < 0.001; Table 1).

## 4 Discussion

In our study, we demonstrated that the overall tissue architecture deterioration of the heart and liver after alcohol exposure was significantly reduced with atorvastatin treatment. Alcohol is known to cause serious damage to the liver and cardiovascular organs. In parallel with our histopathological observations, a previous study modeling alcohol‐related cardiac damage in mice reported that simvastatin—another prominent HMG‐CoA reductase inhibitor—significantly reduced cardiomyocyte hypertrophy, cardiac fibrosis, and inflammation [5], supporting the protective potential of statin therapy against alcohol‐induced structural remodeling.

In our study, we demonstrated that the marked increases in Caspase‐3, Cytokeratin 18‐M30, and Cytokeratin 18‐M65 levels—well‐established biomarkers associated with apoptotic and necrotic cell death—induced by alcohol exposure in serum, heart, and liver tissues were significantly mitigated in the ALCOHOL + hATOR group. Literature regarding the effects of atorvastatin on apoptosis presents a dual nature; while some studies state that statins can induce apoptosis and mitochondrial dysfunction through ROS accumulation under specific toxicological conditions [23, 24], our findings unequivocally demonstrate a potent anti‐apoptotic and cytoprotective role for atorvastatin against alcohol toxicity. To understand this protective mechanism, the direct link between thiol‐disulfide homeostasis, statin pleiotropy, and apoptotic pathways must be elucidated. Atorvastatin's underlying cytoprotection is heavily driven by its cholesterol‐independent pleiotropic effects, which originate from the inhibition of isoprenoid intermediates (e.g., farnesyl pyrophosphate and geranylgeranyl pyrophosphate). This inhibition subsequently prevents the isoprenylation and activation of small GTPases from the Rho family, most notably Rac1 and RhoA. Because Rac1 is an indispensable cytosolic assembly factor for NADPH oxidase, its suppression by high‐dose atorvastatin directly blunts the upstream production of superoxide anions and reactive oxygen species (ROS). Furthermore, this pleiotropic cascade facilitates the preservation and activation of endothelial nitric oxide synthase (eNOS) via Akt‐dependent phosphorylation, which aids in maintaining microvascular perfusion and preventing reactive nitrogen species accumulation. Under severe oxidative stress induced by alcohol, excessive ROS production that is left unchecked oxidizes the functional sulfhydryl (─SH) groups of cellular proteins into disulfide bonds (─S─S─), which is directly reflected in our study by the depleted native/total thiol levels and elevated disulfide levels in the ALCOHOL group. This profound disruption of the redox pool alters mitochondrial membrane permeability, facilitating the opening of the mitochondrial permeability transition pore (mPTP) and the subsequent leakage of cytochrome c into the cytosol. Once in the cytosol, cytochrome c triggers the cleavage and activation of Caspase‐3, leading to the degradation of structural proteins like cytokeratin 18, as evidenced by our high M30 and M65 levels. Crucially, by successfully reversing the Rho/Rac1/NADPH oxidase‐driven pro‐oxidant axis and thereby restoring native and total thiol pools while reducing disulfide levels, high‐dose atorvastatin treatment (ALCOHOL + hATOR) preserves the cellular redox environment. This preservation indirectly maintains downstream mitochondrial membrane integrity, thereby suppressing the apoptotic cascade, as confirmed by the concurrent reduction of Caspase‐3, M30, and M65 in the serum, heart, and liver tissues.

Many studies suggest that various pro‐inflammatory cytokines produced by active Kupffer cells may play a role in the onset of alcoholic liver disease. For example, elevated circulating levels of TNF‐α, IL‐1β, and IL‐6 have been observed in humans and animal models with alcohol‐induced liver damage [24, 25]. In a study by Whang et al. on rats with induced traumatic brain injury, it was found that atorvastatin could provide neuroprotection in traumatic brain injury by stabilizing the integrity of the blood‐brain barrier, reducing inflammation, and mitigating cell death [26]. In our study, we observed that the levels of inflammatory markers IL‐1β, TNF‐α, and HsCRP increased with alcohol consumption and decreased with high‐dose atorvastatin administration. In alcoholism, the increase in TNF‐α levels is indicative of cardiac pathologies, fibrosis, and necrosis [4, 5]. In a study conducted in rats by İto et al., it was found that the combination of atorvastatin with ethanol did not increase the release of liver damage marker enzymes (alanine aminotransferase, aspartate aminotransferase, and lactate dehydrogenase) [27].

In our study, the absence of prominent inflammatory changes, hepatocellular injury, and necrosis despite alcohol exposure is consistent with the staged progression of alcoholic liver disease. It is well established that steatosis represents the earliest and most dominant histopathological feature of alcoholic liver disease whereas inflammation and cellular injury typically emerge in more advanced stages of the disease [28, 29]. In this context, the observed histological findings likely reflect an early‐stage disease phenotype. According to the “two‐hit” hypothesis, steatosis constitutes the first hit that sensitizes the liver to secondary insults; however, the development of steatohepatitis and necrosis requires adequate activation of a second hit involving oxidative stress, endotoxin translocation, and pro‐inflammatory cytokines such as TNF‐α [30]. In our model, this second‐hit process may have remained insufficient. In particular, Sprague‐Dawley rats have been reported to exhibit variable and often limited inflammatory responses to alcohol exposure, and in 28‐day subchronic alcohol models, steatosis remains the predominant and sometimes sole histopathological finding [31, 32, 33]. In addition, insufficient activation of the gut–liver axis and limited TLR4‐mediated signaling induced by endotoxin may have further contributed to the lack of a robust inflammatory response [34]. Taken together, these findings suggest that the observed histopathological pattern is consistent with early‐stage alcoholic liver injury, and that the absence of inflammation and necrosis may be attributed to the limited duration and different characteristics of the animal models [35].

In our study, we showed that although the IMA value increased in the serum of the animals due to alcohol, this increase was significantly reduced with high‐dose atorvastatin. This finding is highly consistent with the literature; for instance, high‐dose atorvastatin was previously shown to alleviate ischemia/reperfusion injury after kidney transplantation in rats by significantly reducing oxidative stress, reactive oxygen species, and inflammation [13]. Our results expand upon these findings by demonstrating that the anti‐ischemic and cytoprotective properties of high‐dose atorvastatin are also highly effective in preserving cardio‐hepatic tissues under alcohol‐induced subchronic toxicity.

Using TAS, TOS, OSI, and MDA analyses, we determined that alcohol‐related oxidative stress was reduced with high‐dose atorvastatin administration. An experimental study conducted on a homogenous group of Wistar rats demonstrated the significant effect of alcohol on oxidative stress and liver inflammatory markers [36]. According to the results of a study by Zamani et al., oxidative damage and inflammation play a critical role in ethanol‐induced hepatotoxicity, and this condition is significantly inhibited by atorvastatin administration. Therefore, atorvastatin has been recommended for the prevention of ethanol‐induced hepatotoxicity [37]. Furthermore, the significance of targeting reactive oxygen species (ROS) in alcohol toxicity has been highlighted by studies exploring diverse protective agents; for example, 6‐gingerol was found to suppress the production of ROS and mitigate alcohol‐related, ROS‐mediated cardiac tissue damage in rats [14]. In alignment with these antioxidant approaches, our study underscores that atorvastatin effectively counters this pro‐oxidant environment. Beyond its well‐known cholesterol‐lowering effects, atorvastatin exerts profound pleiotropic activities, particularly in modulating oxidative stress pathways [11]. Statins exhibit antioxidant properties by inhibiting NADPH oxidase, preventing reactive oxygen species (ROS) generation, and upregulating endogenous antioxidant enzymes [11, 38]. Crucially, recent evidence highlights the role of the Nrf2 (Nuclear factor erythroid 2‐related factor 2) signaling pathway as a major target of atorvastatin‐mediated cytoprotection [38, 39]. Under physiological conditions, Nrf2 is bound to its inhibitor Keap1. Atorvastatin has been shown to induce the dissociation of Nrf2 from Keap1, facilitating its nuclear translocation [39]. Once in the nucleus, Nrf2 binds to Antioxidant Response Elements (ARE) to launch the transcription of key antioxidant and detoxifying genes, including heme oxygenase‐1 (HO‐1) and superoxide dismutase (SOD) [38, 39]. In alignment with these molecular mechanisms, our findings demonstrating that atorvastatin treatment—particularly at high doses—significantly reduced key oxidative biomarkers, namely MDA and OSI, strongly suggest that atorvastatin mitigates alcohol‐induced oxidative tissue injury, at least in part, by activating the Nrf2‐driven antioxidant defense system.

Although atorvastatin treatment demonstrated protective effects against alcohol‐induced cardiac injury, the higher cardiac injury score observed in the high‐dose atorvastatin group suggests that increasing the dose did not provide additional benefit for all histopathological parameters, which may be related to dose‐dependent variations in tissue response and cellular adaptation mechanisms [40].

Although atorvastatin treatment demonstrated protective effects against alcohol‐induced cardiac injury, the higher cardiac injury score observed in the high‐dose atorvastatin group suggests that increasing the dose did not provide additional benefit for all histopathological parameters. This finding may be associated with dose‐dependent alterations in mitochondrial function, oxidative balance, and cellular adaptive responses [41]. Previous studies have shown that atorvastatin can exert cardioprotective effects through the regulation of oxidative stress, apoptosis, and mitochondrial quality control pathways; however, higher doses have also been associated with mitochondrial ultrastructural alterations and impaired mitochondrial respiration in muscle and cardiac tissues

Chronic and excessive alcohol consumption directly toxically affects heart muscle cells, increasing myocyte apoptosis/necrosis, resulting in structural damage, fibrosis, and alcoholic cardiomyopathy. Thiamine (vitamin B1) deficiency, a particular consequence of chronic alcoholism, can disrupt the heart's energy metabolism and lead to heart failure. Chronic alcohol consumption triggers processes such as fatty liver disease (steatosis), inflammation (alcoholic hepatitis), and scar tissue formation (cirrhosis) in the liver [7]. Cholesterol is formed by the conversion of acetyl‐CoA, a substrate, into mevalonic acid by HMG‐CoA reductase. Atorvastatin, by inhibiting the HMG‐CoA reductase enzyme, acts as a rate limiter, reducing cholesterol synthesis in the liver [42]. High cholesterol (LDL) levels increase the risk of heart attack and stroke due to the accumulation of cholesterol‐rich plaques in the artery wall. In addition to its lipid‐lowering effect, atorvastatin is also known to have pleiotropic effects such as improving endothelial function, stabilizing atherosclerotic plaques, and attenuating systemic inflammation. Furthermore, gender‐based variations represent a critical factor in both alcohol‐induced toxicity and statin pharmacokinetics. Clinically and experimentally, females often display lower gastric alcohol dehydrogenase (ADH) activity and distinct volume of distribution, which can accelerate the development of alcoholic tissue injury compared to males. Conversely, endogenous oestrogens are known to exert prominent antioxidant and tissue‐protective effects, which may modulate the baseline susceptibility to subchronic oxidative damage. Regarding the treatment phase, atorvastatin responses can also exhibit sex‐dependent differences, often influenced by variations in cytochrome P450 (particularly CYP3A4) expression and hepatic transporter proteins. In our experimental design, a balanced randomization (4 males and 4 females per group) was strictly maintained to minimize systemic gender bias, providing an essential alignment with contemporary translational research standards.

Translating the findings of experimental animal models to clinical human conditions requires careful consideration of dosing and metabolic differences. In our study, the standard (10 mg/kg) and high (40 mg/kg) doses of atorvastatin were selected based on well‐established rodent protocols. According to the Food and Drug Administration (FDA) guidelines for human equivalent dose (HED) calculation based on body surface area, a rat dose of 10 and 40 mg/kg roughly translates to a human dose of approximately 1.6 and 6.5 mg/kg, respectively. For a 70 kg adult, these doses equal approximately 110 and 450 mg daily. Clinically, the maximum approved daily dose of atorvastatin for humans is 80 mg due to potential risks of myotoxicity and hepatotoxicity. Therefore, while the standard dose used in our study is close to the high‐intensity statin therapy used in clinical practice (e.g., 40–80 mg/day for acute coronary syndromes), the experimental high dose (hATOR) represents a supra‐therapeutic range for humans. Consequently, direct translation of the 40 mg/kg rat dose to human clinical trials is not directly feasible. However, these experimental findings are highly valuable as a “proof‐of‐principle.” They demonstrate that maximizing the activation of the Nrf2 pathway and preserving the thiol‐disulfide balance can effectively halt alcohol‐induced multi‐organ apoptosis (Caspase‐3, M30, M65). Clinically, this suggests that patients already receiving high‐intensity atorvastatin therapy may possess a higher threshold of resistance against alcohol‐induced oxidative tissue damage and underscores the potential for developing target‐specific Nrf2 activators or non‐toxic statin derivatives with enhanced pleiotropic profiles for clinical use.

## 5 Limitations and Future Perspectives

Although this study provides robust biochemical and histopathological evidence regarding the protective effects of high‐dose atorvastatin against alcohol toxicity, certain limitations must be acknowledged. First, while we incorporated Sex as a Biological Variable (SABV) in our design and found no statistically significant differences in primary outcomes between male and female cohorts, the relatively small sample size per sex (*n* = 4) limits the statistical power to detect subtle, sex‐stratified pleiotropic variations. Second, while the 28‐day daily intraperitoneal (i.p.) administration of 20% ethanol successfully established distinct subchronic cardio‐hepatic injury and oxidative stress, this route lacks the physiological translational relevance of chronic oral exposure models, such as the Lieber–DeCarli or NIAAA liquid diets, which better mimic human voluntary alcoholism. Third, our experimental design lacked an independent high‐dose atorvastatin‐only (40 mg/kg) control group to completely isolate the baseline effects of high‐dose statins, which is an important consideration given reports indicating potential dose‐dependent statin hepatotoxicity under non‐challenged conditions. Fourth, the absence of standard serum liver injury markers (ALT, AST, ALP, GGT, total bilirubin), classical cardiac necrosis biomarkers (troponins, CK‐MB, NT‐proBNP), and baseline serum lipid profiles (TC, LDL‐C, HDL‐C, TG) represents a notable limitation that restricts the direct clinical translation of our findings. Furthermore, no functional cardiac assessments, such as echocardiography or electrocardiography (ECG), were performed to evaluate real‐time in vivo myocardial performance. However, our study relied on highly sensitive, mechanistically advanced downstream tissue biomarkers—including Caspase‐3, Cytokeratin 18 (M30/M65), and detailed thiol‐disulfide homeostasis pools—which comprehensively demonstrated that high‐dose atorvastatin exerts a dominant, cell‐preserving, and anti‐apoptotic cytoprotection under an active alcohol challenge. Finally, future longitudinal clinical trials and long‐term animal studies incorporating routine clinical chemistry, functional imaging, oral alcohol diets, and isolated high‐dose statin arms are fully required to validate these distinct interactions.

## 6 Conclusion

In conclusion, our findings demonstrate that subchronic alcohol exposure induces significant histopathological deterioration, lipid peroxidation, inflammatory cytokine responses, ischemia, and apoptotic activation in both rat cardiac and hepatic tissues. Atorvastatin administration mitigates these adverse pathological processes, with the high‐dose treatment (40 mg/kg) displaying a more pronounced protective efficacy than the standard dose (10 mg/kg). This tissue‐protective effect is directly evidenced by the significant reduction in cellular injury scores, suppression of apoptotic markers (Caspase‐3, CK18‐M30, CK18‐M65), and down‐regulation of inflammatory mediators (IL‐1β, TNF‐α, HsCRP, Endothelin‐1). Furthermore, atorvastatin effectively alleviates oxidative stress by lowering TOS, OSI, and MDA levels while successfully restoring TAS and serum thiol‐disulfide homeostasis. Overall, these results indicate that high‐dose atorvastatin serves as a potent agent in restricting alcohol‐induced cardio‐hepatic damage and systemic oxidative imbalance within the biochemical and histopathological limits of the evaluated biomarkers. While these findings suggest a potential protective role through downstream anti‐apoptotic and redox‐preserving pathways, any direct claims regarding the regulation of specific mitochondrial machinery or immediate translational implications for human clinical practice remain speculative at this stage. Further longitudinal studies incorporating direct functional assessments are required to confirm the precise clinical utility of this therapeutic approach.

## Author Contributions

**Cumaali Demirtas:** conceptualization, investigation, methodology, project administration, writing – original draft, data curation. **Şahhan Kılıç:** conceptualization, data curation, methodology. **Mert Babaoğlu:** investigation, methodology. **Süha Asal:** methodology, data curation. **Elif Gökçe Tenekeci:** writing – original draft, writing – review and editing, data curation. **Hakan Beyaztaş:** investigation, data curation. **Eray Metin Guler:** investigation, data curation. **Salime Pelin Erguven:** investigation, data curation. **Kubra Sevgin:** investigation, data curation.

## Funding

The authors have nothing to report.

## Conflicts of Interest

The authors declare no conflicts of interest.

## Data Availability

The data that support the findings of this study are available from the corresponding author upon reasonable request.
